# Biomarkers of sperm fertility in pigs: a relevant tool for the discrimination of sub‑fertile males?

**DOI:** 10.1590/1984-3143-AR2026-0051

**Published:** 2026-09-18

**Authors:** Manuel Álvarez-Rodríguez, Adrián Martín-San Juan, Helena Nieto-Cristóbal, Alejandro Vicente-Carrillo, Eduardo de Mercado

**Affiliations:** 1 Department of Animal Reproduction, Instituto Nacional de Investigación Agraria y Alimentaria, Consejo Superior de Investigaciones Científicas – CSIC, Madrid, España; 2 Departamento de Producción Animal, Facultad de Veterinaria, Universidad Complutense de Madrid, Madrid, España

**Keywords:** extracellular vesicles, receptors, sperm physiology, fertility

## Abstract

In internally fertilizing animals, such as the pig, males transfer not only spermatozoa but also a complex mixture collectively termed seminal plasma (SP). SP contains diverse bioactive components, including proteins, peptides, cytokines, and various RNA species. Growing evidence indicates that SP contributes not only to the sperm quality but also modulates the female’s immune responses. Major progress in biological knowledge and in the discovery of sperm quality biomarkers has been driven by advances in biotechnology and by the increasing affordability of omics technologies. With modern proteomic, (epi)genomic, transcriptomic, metabolomic, and functional analyses applied directly to semen, it is now possible to evaluate not only the functional state of the testes, epididymis, and accessory glands, but also to predict fertility through emerging biomarkers. Beyond diagnostics and fertility forecasting, new opportunities arise from the sperm quality biomarker definition using semen additives—such as specific molecules (proteins, peptides, enzymes) or extracellular vesicles (EVs). These EVs contain bioactive cargo protected within nanovesicles that can be isolated, stored, or even produced de novo *in vitro.* Boar spermatozoa are notoriously difficult to cryopreserve because they lose cholesterol during handling. EVs could fuse with sperm cells, deliver cholesterol‑rich lipids to the sperm membrane, and transfer specific proteins and nucleic acids. This raises the possibility that harvesting EVs, especially from highly fertile males, could be used in the future to improve sperm storage and even cryopreservation. Taken together, this review aims to list sperm ***fertility*** biomarkers that could be useful for the AI companies to discriminate sub-fertile males at an early stage.

## Introduction

Pig production relies heavily on artificial insemination (AI) (Henneberg et al., 2023; Knox, 2016; Maside et al., 2023), for breeding elite animals within nucleus herds and for generating multiplier animals destined for commercial production, so small improvements in boar fertility prediction can have large economic and genetic impacts. Boar spermatozoa arrive in the sow oviduct within minutes after natural mating or AI (Baker and Degen, 1972). They interact with the epithelial lining of the female reproductive tract, triggering molecular and cellular responses with fertility relevance. This interaction supports the establishment and maintenance of pregnancy (Langendijk et al., 2005), elicits an inflammatory reaction in the female tract (Rodriguez-Martinez et al., 2009), and modulates the expression of immune‑related genes (Alvarez-Rodriguez et al., 2019; Álvarez-Rodríguez et al., 2020; Gardela et al., 2020; Ruiz-Conca et al., 2020), ultimately contributing to the development of immune tolerance to paternal antigens (Robertson and Sharkey, 2001) and allowing potentially fertile sperm to survive in the sperm oviductal reservoir (Rodriguez-Martinez et al., 1990). Semen promotes the recruitment of antigen‑presenting cells that process and present paternal antigens to lymphocytes, activating adaptive immune pathways that contribute to immune tolerance during subsequent embryo development (Robertson et al., 2003). Overall, transcriptomic alterations in the porcine uterus and oviduct following exposure to semen have been documented (Alminana et al., 2014; Alvarez-Rodriguez et al., 2019, 2020b). Therefore, this bidirectional communication appears to be initiated by signals derived from SP components, such as proteins, cytokines, and EVs, as well as from spermatozoa themselves, including associated proteins, exosomes, and RNAs (Alvarez-Rodriguez et al., 2019).

The question that arises from the aforementioned male-female cross-talk is, ***how do we isolate the individual effect of the male fertility from the whole reproductive performance?*** Undoubtedly, boar fertility is influenced in part by semen quality, alongside other physiological factors essential for successful reproduction (Rodriguez-Martinez et al., 2024), including *in vitro* embryo production (Chen et al., 2021; Garcia-Canovas et al., 2024). Yet, conventional semen evaluation includes sperm motility, sperm viability, sperm morphology, analysis of subcellular structures such as the acrosome or metabolite concentrations such as intracellular calcium concentrations, etc. (Ax et al., 2000; Björndahl and Brown, 2022) which still explains only a modest fraction of variation in field fertility (Llavanera, 2024). This has driven an intense search for more informative sperm morphological, functional and molecular biomarkers that better predict fertilizing ability, robustness to preservation, and embryo development.

A key requirement for a genetically selected stud boar is the ability to produce and ejaculate large quantities of sperm that possess the characteristics needed for high fertility, two traits that do not always show a positive correlation (Rodriguez-Martinez et al., 2024). Commercial pig production has greatly benefited from the extensive use of AI with semen from genetically selected boars. These males undergo stringent reproductive evaluations to ensure they consistently produce ejaculates containing high numbers of viable and motile spermatozoa (Broekhuijse et al., 2011). However, 5–10% of highly selected breeding boars exhibit fertility outcomes below the breed average and are defined as sub-fertile (Roca et al., 2015; Rodriguez-Martinez et al., 2024): e.g. in a 1500 male farm, approximately up top 150 males per year are potentially sub-fertile, resulting in annual financial losses for the company of approximately €150,000. This unexplained subfertility has long underscored the need for more advanced semen‑analysis strategies to identify and exclude clearly sub-fertile sires (Foxcroft et al., 2010). Given that spermatozoa carry structural and functional attributes that can retrospectively reflect a boar’s fertility potential (Foxcroft et al., 2010), new analytical approaches have increasingly incorporated sperm ‘omics technologies (Kumaresan et al., 2020; Rodriguez-Martinez, 2014, 2013) and SP profiling (Pérez-Patiño et al., 2018). In fact, accurately predicting fertility in either species remains difficult, largely because defining a male’s fertility level requires accounting for numerous confounding variables. These include the number of sperm used for insemination, mating or insemination frequency, and in pigs, additional factors such as the number of females inseminated, the number of inseminations per oestrus, seasonal influences, oestrus‑detection methods, farm conditions, and specific AI procedures. All of these elements must be rigorously and statistically evaluated, together with comparisons within breeding lines, under the assumption that genomic factors influence fertility (Broekhuijse et al., 2012a).

A variety of methods and protocols have been developed to assess boar semen quality (Maside et al., 2023), including the identification and application of molecular biomarkers (Llavanera, 2024; Sutovsky et al., 2024). More recently, the incorporation of omics‑based approaches encompassing genomic variants, proteins, metabolites, and non‑coding RNAs, has further advanced the characterization of sperm quality (Cheng et al., 2024; Khan et al., 2024; Park et al., 2023b; Sá et al., 2025b).

Taken together, this review aims to list sperm ***fertility*** biomarkers (***Figure 1***) that could be useful for the AI companies to discriminate sub-fertile males at an early stage.

**Figure 1:**
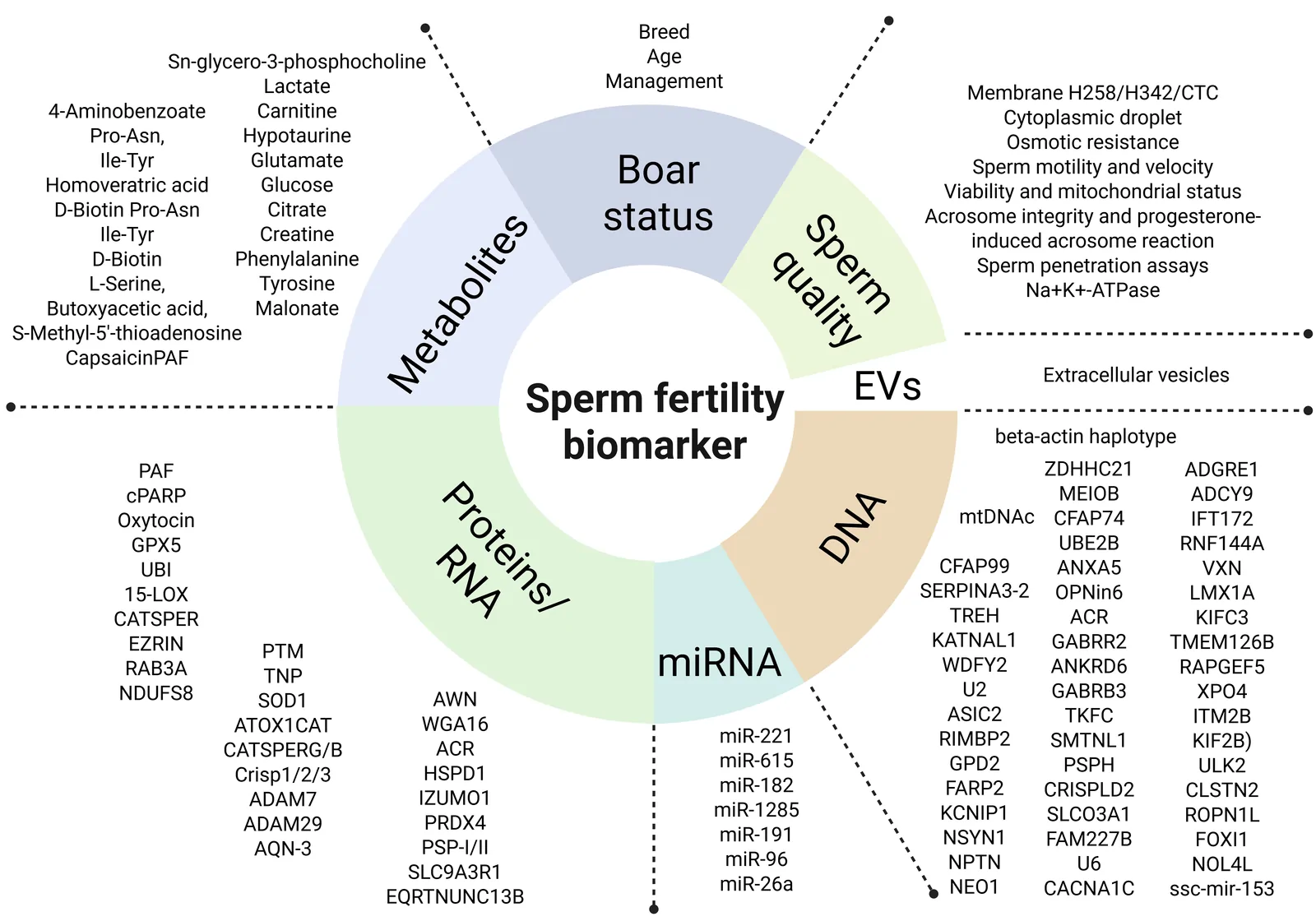
A graphical representation of an up‑to‑date list of sperm fertility biomarkers in the pig, covering aspects from boar management to sperm physiology, metabolites, DNA, and RNA cargo in sperm and/or seminal plasma.

## Sperm fertility biomarkers

Much effort is being made to establish relationships between the molecular events that take place in spermatozoa under fertilizing conditions and actual sperm function during fertilization. The long and complex journey starts as early as in the testis, with crucial hormones such as testosterone (Brinke et al., 2021), anti-Müllerian hormone (Barranco et al., 2020), and oxytocin (Martínez-Hernández et al., 2026). Overall, steroidogenesis in adult Leydig cells is fundamental for several reproductive processes, such as the initiation and maintenance of spermatogenesis, sperm maturation in the epididymis, proper accessory gland function, and the modulation of sexual drive (Desaulniers et al., 2026). The epididymis synthesizes key factors required for successful maturation, fertilization and functions as a reservoir for spermatozoa until ejaculation (Rodriguez-Martinez et al., 2024). These reproductive roles are carried out within the mesonephric‑derived duct system, which is lined by absorptive and secretory epithelial cells capable of merocrine and apocrine release of proteins, antioxidants, electrolyte/pH‑regulating enzymes, and small non‑coding RNAs (sncRNAs). Many of these molecules are packaged into epididymosomes, which mediate their transfer to spermatozoa and contribute to long‑lasting modifications of sperm function (Rodriguez-Martinez et al., 2022). In addition, boar sperm proteome undergoes substantial remodelling during ejaculation, involving proteins with well‑established roles in sperm function (Pérez-Patiño et al., 2019). Then, a selective mechanism likely ensures that only fully mature and structurally intact spermatozoa are capable of effectively interacting with and fertilizing the oocyte. The perivitelline space seems to contribute to sperm surface remodelling (Tsai and Gadella, 2009), as acquisition of CD9 by the mouse sperm may facilitate the successful penetration of the first spermatozoon reaching the oocyte (Barraud-Lange et al., 2007a), being the sperm-egg fusion is mediated by vesicles containing CD9 that are released from the egg and interact with sperm (Miyado et al., 2008). Furthemore, a CD9 independent way seems to appears that does not supply the fertilising ability of Cd9-deleted oocytes (Barraud-Lange et al., 2012). Moreover, also in mice, alpha6beta1 integrin is expressed by both gametes and is functional in their membranes interaction (Barraud-Lange et al., 2007b). The need for survival in the female tract may require much slower sperm responses than are considered optimal for *in vitro* fertilization (Harrison, 1997).

A key priority for the swine industry in improving boar fertility assessment is the validation of laboratory‑based semen quality metrics against *in vivo* reproductive performance (Flowers, 2009). A thorough understanding of the selection criteria and molecular biomarkers that regulate boar reproductive potential is crucial for designing effective breeding strategies aimed at improving reproductive efficiency in swine (Hensel et al., 2024). Overall, direct analysis of crucial field-related parameters in boar samples could shed light on the multifactorial components of male fertility. For example, data from AI centres in a large‑scale field study revealed a positive association between the hypotonic resistance of ejaculates and *in vivo* fertility (Druart et al., 2009). An additional parameter to consider is the variation among boar breed lines, being dependent on the ability of extended semen to maintain motility during storage, as well as in corresponding farrowing rates (Sonderman and Luebbe, 2008). Furthermore, it seems that neither sperm concentration nor the presence of bacteria had any detectable influence on farrowing rate, but sperm concentration influenced the total born in that study (Reicks and Levis, 2008), Additionally, although studies directly linking bacteriospermia or microbiome composition to fertility variables are lacking, it is recognized that an excess of bacteria is detrimental to sperm quality and, therefore, fertility. Truly contaminated doses are removed, and antibiotics usually keep bacterial growth at bay, e.g. a recent study found a negative association between highly abundant Pseudomonas with sperm quality and reproductive potential (Zhang et al., 2020).

Additionally, the development of *in vitro* approaches that mirror, at least in part, the fine tune cross talk between the male and the female counterparts is also relevant. Thus, some studies indicate that an *in vitro* fertilization (IVF) system provides a suitable platform for assessing the quality of frozen–thawed boar semen before its commercial use (Sellés et al., 2003). Results from the sperm penetration assay using zona‑free oocytes showed a strong correlation with historical average litter size, providing markedly improved sensitivity for detecting both low and high fertility outcomes in terms of litter size (Oh et al., 2010). A positive correlation was observed between zona‑binding capacity and fertility when measured by average litter size (r = 0.64, P < 0.05), whereas no such relationship was detected when fertility was assessed by farrowing rate (r = –0.28) (Braundmeier et al., 2004). Thus, zona‑binding capacity alone could not be a completely reliable predictor of fertilizing competence, but combined with additional sperm functional assessments, it can contribute to more accurate fertility predictions (Collins et al., 2008). Other approaches, such as the sperm–oviduct binding assay, evaluate multiple functional attributes of the sperm plasma membrane and may serve as a valuable *in vitro* tool for identifying sub-fertile boars (Waberski et al., 2005). Among the evaluated seminal tests, the homologous *in vitro* penetration (hIVP) performed best and may substantially improve the *in vitro* evaluation of sperm fertilizing capacity (Gadea et al., 1998; Matás et al., 1996). It also decreased the number of sperm tightly bound to the zona pellucida, but increased the proportions of capacitated and acrosome‑reacted sperm, while heparin alone did not enhance capacitation (Kim et al., 1997). Despite the improvements, hIVP) it is not currently widely used.

## Sperm quality

### Sperm motility and kinetics

Computer‑assisted semen analysis (CASA) for sperm motility originated in the late 1970s–1980s, when advances in video microscopy, digitization, and computer tracking algorithms made automated sperm‑motion quantification possible, with the first major commercial system (CellSoft) released in 1985, followed by Hamilton‑Thorne systems in the late 1980s (Amann and Katz, 2004). CASA offers an objective evaluation of multiple sperm kinetics parameters. However, most sperm assays measure only isolated aspects of the fertilization process. Consequently, combining a targeted set of complementary sperm tests yields more accurate predictions of fertilizing capacity than relying on any single assay (Jung et al., 2015). Traditional post‑thaw evaluations of motility, viability, and acrosome integrity were performed for each ejaculate, along with assessments of *in vitro* sperm–oviduct binding and competitive zona‑binding assays, fertilization, cleavage, and blastocyst formation. In this regard, a regression model predicting the proportion of litters sired by each boar was highly robust and incorporated key variables: the proportion of acrosome‑compromised sperm, the percentage of live sperm, total motility, and the number of zona‑bound sperm (Daigneault et al., 2015). Significant associations between sperm motility and male fertility outcomes have been reported across multiple studies in the porcine species (Broekhuijse et al., 2012a, 2012b; Gadea et al., 2004; Tardif et al., 1999). However, only a limited number of studies have examined direct correlations between specific sperm traits and male fertility (Gadea, 2005). For instance, total sperm motility, the proportion of swimming spermatozoa in an ejaculate, has been linked to sperm fertilizing ability (Broekhuijse et al., 2012b; Gadea, 2005). Nevertheless, sperm velocity emerged as a central biomarker and has been strongly linked to mitochondrial function, which underpins motility, capacitation, DNA integrity, and early embryo development, all essential determinants of fertilization efficiency and embryo viability (Toledo-Guardiola et al., 2025).

### Sperm physiology

Flow cytometry has become a key methodology for sperm analysis, and its use continues to expand in both routine semen evaluation and research applications within veterinary reproductive science (Martinez-Pastor et al., 2010)**.** In terms of sperm viability, acrosomal damage would be a reliable predictor of reproductive outcomes in terms of litter size (Holt et al., 1997) and total number born, including in the latter the analysis of cleaved poly-ADP ribose polymerase 1 (cPARP) as a potential novel biomarker of born alive (Ausejo-Marcos et al., 2025). Moreover, the proportion of progesterone‑induced acrosome reactions (IAR) was significantly lower in sub-fertile boars than in fertile boars (Herrera et al., 2002). Combined H33258/CTC staining to study capacitation ability, may serve as a useful alternative approach for estimating male fertility until fertility‑associated biomarkers are more fully validated (Kwon et al., 2018). An increase in fluorescence intensity of membrane‑ruptured H33258‑stained sperm and intact H33342‑stained sperm in boar AI doses was associated with reduced litter size (Sutkeviciene et al., 2009). An osmotic resistance test alongside sperm viability, morphology, and acrosome integrity, together with conventional semen parameters, can enhance the prediction of an ejaculate’s fertilizing potential (Yeste et al., 2010). β‑actin haplotypes association analysis was significantly linked to variation in sperm motility, abnormal sperm rate, and in the number of piglets born alive (Lin et al., 2006b). Previous studies indicate that platelet‑activating factor is present in boar spermatozoa, and its concentrations are significantly higher in males exhibiting high farrowing rates and greater numbers of total and live‑born piglets (Roudebush and Diehl, 2001).

### Mitochondrial status

It is also relevant that sperm function, mitochondrial activity, and *in vivo* fertility are associated with their mitochondrial DNA content (mtDNAc) in pigs. Samples with lower mtDNAc showed higher conception and farrowing rates, but similar *in vitro* fertilization rates and embryo development, when compared to those with greater mtDNAc (Llavanera et al., 2024). Another study tested the combination of sperm motility and ATP concentration, and their relationship with the total number of piglets born (TNB) following AI with Norwegian Landrace (NL) and Norwegian Duroc (ND) boar semen. In NL, TNB was influenced by sperm linearity at collection and wobble after storage, whereas in ND, TNB was affected by the proportion of motile sperm, curvilinear velocity, and lateral head amplitude at collection, as well as linearity following storage (Tremoen et al., 2018).

### Morphological abnormalities

Morphological abnormalities such as cytoplasmic droplets have also been negatively correlated with farrowing rate, although they did not influence the total number of piglets born (Lovercamp et al., 2007a). Correlation analyses identified four independent sperm‑quality traits as significant predictors of boar fertility: proximal cytoplasmic droplets, active mitochondria, beat‑cross frequency of progressively motile spermatozoa, and the oscillation parameter of the actual path (Schulze et al., 2013).

### DNA fragmentation

The origin of sperm DNA‑fragmentation analysis as a scientific field traces back more than 70 years, but the first true diagnostic assays emerged in the 1980s–1990s (Rex et al., 2017). Controversial findings arise from results that fail to correlate with fertility (Batista et al., 2016). However, other studies confirm that 81.7% of the variability in farrowing rates comes from this fragmentation (Tsakmakidis et al., 2010). Moreover, sperm DNA damage affects farrowing rate (Didion et al., 2013, 2009) and litter size in porcine (Boe-Hansen et al., 2008; Boe-Hansen and Satake, 2019; Myromslien et al., 2019), and compromises embryo development, but not oocyte fertilisation (Mateo-Otero et al., 2022a). Overall, understanding which semen traits contribute to male fertility and the magnitude of their contribution, enhances the ability to predict field fertility outcomes (Broekhuijse et al., 2012).

### Sperm molecular fertility biomarkers

Major progress in biological knowledge and in the discovery of sperm quality biomarkers has been driven by advances in biotechnology and by the increasing affordability of omics technologies. With modern proteomic, (epi)genomic, transcriptomic, metabolomic, and functional analyses applied directly to semen (spermatozoa and SP components), it is now possible to evaluate not only the functional state of the testes, epididymis, and accessory glands, but also to predict fertility through emerging biomarkers.

### Transcriptome and proteome

An example is that Zinc-associated molecular patterns may function as mechanistic indicators of fertility within a translational boar model, offering relevance for both livestock breeding and human assisted‑reproduction contexts (Rodriguez et al., 2026). Another example, Glutathione peroxidase‑5 (GPX5), an H_2_O_2_‑scavenging enzyme broadly expressed throughout the boar reproductive tract, shows variable abundance in SP, and higher levels have been positively associated with fertility outcomes in liquid‑stored AI doses (Barranco et al., 2016), including farrowing rate (Novak et al., 2010). Flow‑cytometry analysis of Ubiquitin (UBI) and Arachidonate 15‑lipoxygenase (15‑LOX) exhibited seasonal fluctuations that paralleled seasonal changes in farrowing rate and total number of piglets born. UBI levels were positively correlated with farrowing rate (r = 0.31; P < 0.05), and negatively correlated with total number born (r = –0.38; P < 0.01). In contrast, 15‑LOX values were negatively associated with total number born (r = –0.33; P < 0.05) (Lovercamp et al., 2007b). Additional marked differences in biomarker expression were observed between fertility‑classified boars: some factors were upregulated exclusively at the protein level (catalase [CAT], superoxide dismutase 1 [SOD1], and glutathione‑related proteins), whereas others showed increased expression only at the mRNA level (ATOX1, Antioxidant Protein 1). Moreover, protamines 2 and 3, key for sperm DNA condensation, and the transition proteins TNP1 and TNP2 required for histone‑to‑protamine replacement were overexpressed in spermatozoa from high‑fertility boars (Alvarez-Rodriguez et al., 2021).

It has also been described that the presence of 347 up-regulated and 174 down-regulated RNA transcripts in high-fertility breeding boars, based on differences of farrowing rate (FS) and litter size (LS), relative to low-fertility boars in the AI program (Alvarez-Rodriguez et al., 2020a). Among the plethora of RNA transcripts found, CATSPERG (catSper channel auxiliary subunit gamma) was up‑regulated, whereas CATSPERB (catSper channel auxiliary subunit beta) was down‑regulated in boars with high fertility. Both transcripts encode auxiliary components of the CatSper channel, which play a central role in boar sperm motility during *in vitro* capacitation (Vicente-Carrillo et al., 2017). The CATSPER complex incorporates CATSPERB and CATSPERG, the latter interacting with CATSPER1 (Wang et al., 2009). Inhibition of this channel has been associated with elevated reactive oxygen species (ROS) production (Ghanbari et al., 2019) and, through its Ca^2+^‑dependent signalling functions, it participates in key steps of mammalian fertilization (Ren and Xia, 2010).

The ADAM family of matrix metalloproteinases participates in diverse functions, including cell adhesion and immune‑related signalling pathways, such as the activation of TNF‑α and the generation of active Epidermal Growth Factor Receptor (EGFR) isoforms (Edwards et al., 2009). Previous works also highlighted the relevance of this gene family in sows, particularly in relation to cellular adhesion processes and the pH regulation of the utero‑tubal junction (Atikuzzaman et al., 2017, 2015). Moreover, ADAM7 (ADAM metallopeptidase domain 7) and ADAM29 (ADAM metallopeptidase domain 29) were down‑regulated in high‑fertility boars. ADAM7 is required for normal fertility in mouse sperm (Choi et al., 2015), as both ADAM proteins have been associated with enhanced adhesion to extracellular matrix components (Wei et al., 2011), with a potential influence on sperm progression through the female reproductive tract remaining (Alvarez-Rodriguez et al., 2020a).

Additional studies of mRNA levels suggested the fertility-related biomarkers HSPD1, IZUMO1, PRDX4, PSP‑I, and SLC9A3R1 (r = −0.44, −0.51, 0.58, 0.52, and −0.71, respectively) and the sperm motility biomarkers, all positively correlated, HSPD1, EQTN, UNC13B, PSP-I, and PSP- II (Pang et al., 2023). Two of them, PSP‑I and PSP‑II, have been previously found in spermatozoa and differed significantly between boars with high and low litter sizes (Kang et al., 2019). One of the mentioned spermadhesins, PSP-I, and AWN have been proposed to play roles in preventing premature capacitation within the sow uterus (Caballero et al., 2009; Vadnais and Roberts, 2010). Notably, PSP‑I has also been linked to reduced fertility outcomes (farrowing rate) (Novak et al., 2010). An additional marker showing a positive correlation with farrowing rate was PRDX4 mRNA expression (Pang et al., 2023), as previously stated for its highest accuracy for male fertility prediction and diagnosis (Ryu et al., 2021) and an accuracy of 85% for predicting male fertility by the SLC9A3R1 gene (Pang et al., 2023), a biomarker suggested to effectively predict fertility replacing conventional motility parameters and capacitation status (Kim et al., 2019).

### Micro RNA-ome

Regarding non-coding signalling, such as miRNAs, small molecules present in SP, two microRNAs (miRNAs) showed differential expression in high‑fertility boars: miR‑615 was up‑regulated, whereas miR‑221 was down‑regulated. miR‑615 is known to inhibit apoptosis through targeting EGFR (Qiu et al., 2018), and its activity has been documented in sperm in association with epidermal growth factor receptor (EGFR) signalling, suggesting a potential link between capacitation and acrosome reaction dynamics and miR‑615 expression (Michailov et al., 2014). In contrast, miR‑221 has been associated with Wnt2, BDNF, and CREB‑related genes (Lian et al., 2018), aligning with previous findings showing down‑regulation of CREB3L2 in the uterus and utero‑tubal junction of sows following both mating and AI. Additionally, miR‑221 is connected to the PI3K–Akt and oestrogen signalling pathways (Alvarez-Rodriguez et al., 2019). An additional set of four miRNAs are differentially expressed between high-fertility and low-fertility boars: mir-182, mir-1285, mir-191, and mir-96, with pivotal roles in sperm survival and immune tolerance (Martinez et al., 2022). Analysis of boar ejaculates identified a distinct profile of EV–associated miRNAs, with 44 miRNAs enriched in EVs from high‑fertility boars, whereas nine were less abundant compared with low‑fertility animals (Chen et al., 2024). Notably, the elevated expression of miR‑26a in the high‑fertility group is proposed to influence sperm physiology through regulation of HMGA1, exerting measurable inhibitory effects on sperm viability, motility, acrosomal integrity, plasma membrane stability, and ATP content (Chen et al., 2024).

### Metabolome

Metabolome, firstly defined in 1998 (Oliver et al., 1998) refers to the complete set of small‑molecule metabolites (e.g., metabolic intermediates, hormones, signaling molecules, secondary metabolites) present in a biological sample. Metabolites play pivotal roles in diverse biological processes, although metabolomic approaches in porcine SP in relation to *in vivo* fertility are still scarce, but with promising results as fertility biomarkers (Mateo-Otero, 2024). Some studies showed that 4-Aminobenzoate, Pro-Asn, Ile-Tyr, Homoveratric acid, Ile-Tyr, and D-Biotin were over-represented in boar semen with high conception rate index, whereas L-Serine, Butoxyacetic acid, S-Methyl-5'-thioadenosine, Capsaicin and 1-O-(cis-9-Octadecenyl)-2-O-acetyl-sn-glycero-3-phosphocholine (PAF) were under-represented in boar semen with high conception rate index (Zhang et al., 2021). An additional study suggested increased lactate levels as a farrowing rate fertility biomarker, while carnitine, hypotaurine, sn-glycero-3-phosphocholine, glutamate, glucose levels as litter size fertility biomarkers (Mateo-Otero et al., 2021). In addition, a positive correlation was found among citrate, creatine, phenylalanine, tyrosine, and malonate levels in relation to stillbirths per litter, and malonate and fumarate levels in relation to gestation length (Mateo-Otero et al., 2021).

Hormones also seem to play an important role. The SP concentration of oxytocin was influenced by boar identity, ejaculate, and age, and showed a positive association with both ejaculate volume and the farrowing rates of liquid‑stored semen AI doses (Padilla et al., 2021). Indeed, porcine seminal EVs present oxytocin on their external surface, with those derived from accessory sex glands showing marked enrichment. This enrichment is associated with increased farrowing rates but may reduce litter size, consistent with its influence on myometrial contractility, which can promote sperm transport but may interfere with optimal embryo implantation (Parra et al., 2025).

### Secretome, extracellular vesicles and its cargo

Nowadays, the secretome, the full repertoire of proteins secreted by a biological system, including both classical and non‑classical secretion pathways (Tjalsma et al., 2000). These proteins, both free and inside EVs contained in boar SP play key regulatory roles in sperm capacitation, acrosome reaction, and motility (Rodriguez-Caro et al., 2019), sometimes inhibiting sperm acrosome reaction and *in vitro* fertility, with a negative correlation with the EZRIN protein, an active protein involved in EVs-sperm interactions (Xu et al., 2024). Notably, EVs are selective carriers of biomolecules that promote fertilization through a fine-tune modulation of the female genital tract (Rodriguez-Martinez and Roca, 2022). Indeed, the EV proteome differs between higher and lower fertile boars, with many of the differentially expressed proteins known to be involved in reproductive processes (Barranco et al., 2026). It is also relevant that a high total antioxidant capacity in porcine seminal plasma (SP‑TAC) is associated with enhanced sperm survival and fertility (Barranco et al., 2015). The SP effect has even been used to improve conception and farrowing rates when used as a supplement in AI extenders (Rozeboom et al., 2000). Among thousands of proteins in the SP, carbohydrate-binding protein AQN-3 (AQN3), AQN1, wheat germ agglutinin 16 (WGA16), acrosin (ACR), heat shock protein 90 (HSP90), and cysteine-rich secretory protein 1–2 (CRISP1–2) are positively correlated to high fertile boars (Zeng et al., 2021), with functions related to sperm membrane integrity and binding to the zona pellucida, and CRISP2 directly related to boar fertility (Gao et al., 2021). Another family member, CRISP3, is enriched in SP and localized in the post-acrosomal region of the sperm head, migrating to the tail in response to capacitation, and with immunomodulatory action by decreasing IL-α, IL-1β, and IL-6, and potentially regulating the female reproductive tract inflammatory response (Bu et al., 2024). The Acidic Quiescent Non‑glycosylated spermadhesin 3 (AQN-3) and seminal plasma motility inhibitor (SPMI), which share high homology and are also associated with capacitated sperm, are increased in low-litter-size boars, thus negatively linked to fertility (Kwon et al., 2015; Novak et al., 2010). In contrast, some specific isoforms of Na+/K+-ATPase in the sperm head are correlated to boar *in vivo* fertility, probably through Na+⁄K+-ATPase's role in capacitation (Imran et al., 2025). Another relevant family of proteins involved in membrane and EVs transport is the Rab family, expressed in the acrosomal region and in the tail of the spermatozoa. These proteins reduced their expression in response to *in vitro* capacitation, suggesting a pivotal role in sperm quality prediction (Bae et al., 2019), such as Rab3A for its correlation to sperm motility and kinematic parameters (Jang et al., 2024), but also as a sperm fertility biomarker (Bae et al., 2022), such as Rab2A (Kwon et al., 2017). A relevant set of mitochondrial proteins has emerged as litter-size predictors, as in the case of NDUFS8 (Lee et al., 2023). Other SP proteins positively correlated with farrowing rate (FURIN, AKR1B1, UBA1, PIN1, SPAM1, BLMH, SMPDL3A, KRT17, KRT10, TTC23, and AGT) and litter size (PN-1, THBS1, DSC1, and CAT) have been found. However, FURIN, sperm adhesion molecule 1 (SAPM1) (related to farrowing rate), Nexin-1 and CAT (related to litter size) are the most promising fertility biomarkers (Pérez-Patiño et al., 2018). On the other hand, the Aldo-Keto Reductase Family 1 Member B (AKR1B1), was negatively correlated with *in vitro* embryo production (Mateo-Otero et al., 2022b). In addition, two ciliary-related proteins, the dynein axonemal light intermediate chain 1 (DNALI1) and the radial spoke head component 9 (RSPH9), are also suggested as male fertility predictors (Bae et al., 2024).

### Genome-Wide Association Study (GWAS)

Genome-Wide Association Study (GWAS) applied to sperm and semen traits began in the late 2000s, and the field expanded rapidly after 2015, with large‑scale boar datasets enabling the first robust QTL discoveries for motility, morphology, and sperm production traits (Gòdia et al., 2020). Studies using Genome-Wide Association Study (GWAS) provide novel insights into semen quality traits in mammals, identifying 234 candidate loci associated with semen quality for pig selective breeding (Lin et al., 2026). In addition, GWAS for gestational traits in the Danish Landrace pig population revealed 13 significant genes that are also under selection in selective sweeps. These genes include INSYN1, NPTN, NEO1, and ZDHHC21 (Chen et al., 2025b). E.g., NPTN is essential for fertility in mice (Chen et al., 2023) and ZDHHC21 is associated with conception rate in cows (Kiser et al., 2019). Six missense variants have been identified in high linkage disequilibrium (LD) with lead SNPs in genes related to sperm production (e.g., MEIOB, CFAP74, and UBE2B) (Sá et al., 2025a). that have also been described in humans with azoospermia (Gershoni et al., 2017), asthenoteratozoospermia (Chen et al., 2025a), and oligozoospermia (Yatsenko et al., 2013), respectively. AnnexinA5 (ANXA5) has been identified as a positional candidate gene for reproduction and fertility traits in boars and contributes to the growth of immature Sertoli cells (Han et al., 2025). Moreover, the fat mass and obesity-associated protein (FTO)–ANXA2 regulatory pathway therefore constitutes a promising molecular target for strategies aimed at alleviating heat‑stress–induced reproductive impairment and safeguarding male fertility (Li et al., 2026). Other candidate biomarker genes were OPNin6, related to the number of piglets born alive, and acrosin (ACR), affecting non-return rate (Lin et al., 2006a). Additional candidate SNPs identified in relation to boar fertility are gamma-aminobutyric acid type A receptor subunit rho2 (GABRR2), ankyrin repeat domain 6 (ANKRD6), gamma-aminobutyric acid type A receptor subunit beta3 (GABRB3), triokinase and FMN cyclase (TKFC), smoothelin like 1 (SMTNL1), phosphoserine phosphatase (PSPH), cysteine rich secretory protein LCCL domain containing 2 (CRISPLD2), solute carrier organic anion transporter family member 3A1 (SLCO3A1), alpha-1-antichymotrypsin 2 (SERPINA3-2), cilia and flagella associated protein 99 (CFAP99), trehalase (TREH), katanin catalytic subunit A1 like 1 (KATNAL1), WD repeat and FYVE domain containing 2 (WDFY2), U2 spliceosomal RNA (U2), acid sensing ion channel subunit 2 (ASIC2), RIMS binding protein 2 (RIMBP2), glycerol-3-phosphate dehydrogenase 2 (GPD2), FERM, ARH/RhoGEF and pleckstrin domain protein 2 (FARP2), potassium voltage-gated channel interacting protein 1 (KCNIP1) (Pértille et al., 2021).

### Methylome

At the DNA level, the methylome, the complete set of DNA methylation marks (primarily 5‑methylcytosine, but also other modified cytosines) across the entire genome (Zhang et al., 2006) has arisen as a promising fertility biomarker, identifying both conserved and species‑specific methylation patterns, as well as altered methylation at the GNAS locus in infertile boars (Congras et al., 2014). Moreover, it shows a strong correlation with fertility (farrowing rate and litter size), with interesting findings on differentially methylated regions mostly hypermethylated in late summer compared to mid-autumn (Pértille et al., 2021). The combination of Genotyping By Sequencing (GBS) and methylated DNA immunoprecipitation (MeDIP) allows the simultaneous identification of genetic variation through Genomic-Wide Association Studies (GWAS) and epigenetic differences in Differentially Methylated Regions (DMR). Notably, hypermethylated regions in low-fertile boars have been identified in genes such as thymocyte selection associated (THEMIS), family with sequence similarity 227 member B (FAM227B), U6 spliceosomal RNA (U6), calcium voltage-gated channel subunit alpha1 (CACNA1C), adhesion G protein-coupled receptor E1 (ADGRE1), adenylate cyclase 9 (ADCY9), intraflagellar transport 172 (IFT172), ring finger protein 144A (RNF144A), vexin (VXN), LIM homeobox transcription factor 1 alpha (LMX1A), kinesin family member C3 (KIFC3), transmembrane protein 126B (TMEM126B), Rap guanine nucleotide exchange factor 5 (RAPGEF5), exportin 4 (XPO4), integral membrane protein 2B (ITM2B), kinesin family member 2B (KIF2B), U6 spliceosomal RNA (U6), unc-51 like autophagy activating kinase 2 (ULK2), calsyntenin 2 (CLSTN2), rhophilin associated tail protein 1 like (ROPN1L), forkhead box I1 (FOXI1), COMM domain containing 7 (NOL4L), and ssc-mir-153 (Pértille et al., 2021).

### Artificial intelligence fertility predictors

The integration of artificial intelligence and machine learning is revolutionizing reproductive management in boar studs by introducing unbiased, data‑centric approaches for assessing and forecasting sperm quality (Hensel et al., 2026). Convolutional neural networks trained on image‑derived flow cytometry outputs or CASA datasets have demonstrated high precision in quantifying sperm morphological features and detecting acrosomal abnormalities (Keller et al., 2025). Learning vector quantization applied to automatic classification of the acrosome status of boar spermatozoa has been successfully developed (Alegre et al., 2008), complemented by the Acrosome Reaction Classification System developed recently (Park et al., 2023a). It has been reported the first indication that the sperm mobility assay, adapted for extended boar semen doses, can partially discriminate between high‑ and low‑fertility males and reduce classification error when combined with CASA‑derived parameters (Mills et al., 2026). Two‑step clustering approach as a straightforward and effective method for identifying boars with differing fertility levels, offering a novel framework for quantifying boar fertility, mainly by average total litter size, number of piglets born alive, and number of healthy piglets (Huang et al., 2023). Moreover, t-distributed stochastic neighbour embedding (t‑SNE) analysis uncovered a hierarchical structure in sperm motility patterns both within and across ejaculates, enabling robust fertility prediction through Bayesian logistic regression, indicating that motility characteristics, particularly high‑velocity and linear trajectories, are positively associated with fertility and contribute more strongly to predictive accuracy than other sources of variation (Fernández-López et al., 2022). Multivariate analyses revealed that sperm parameters correlate with systemic physiological markers (e.g., organ function, oxidative stress, lipid and carbohydrate metabolism), reinforcing the idea that sperm quality integrates broader male health signatures relevant to sperm quality biomarkers reflecting not only fertilizing capacity, but also paternal contributions to early-life robustness (Toledo-Guardiola et al., 2025). Integrating assessments of fresh‑ejaculate motility with freezability measures, including a thermoresistance test, may improve the effectiveness of boar selection strategies (de Mercado et al., 2025). Finally, artificial intelligence offers substantial value for semen assessment by reducing operator‑dependent variability, improving analytical throughput, and enabling the development of new label‑free diagnostic approaches, contributing to address major constraints that currently limit the broader application of molecular biomarkers in semen analysis, thereby paving the way for more robust and widely accessible indicators of sperm quality (Keller et al., 2025). Overall, future development of this powerful tool should be applied for identifying reliable sperm fertility biomarkers in pigs, enabling more accurate prediction of reproductive performance and, therefore, aiming to improve the efficiency of swine breeding programs.

## Conclusions and future perspectives

This review attempted to list **fertility biomarkers,** including classical and modern proteomic, (epi)genomic, transcriptomic, metabolomic, and functional analyses applied directly to semen, and whether some of them could be used as **sperm fertility biomarkers**. In this regard, equally important is the ability to accurately estimate relative fertility among boars and to reliably identify and exclude, as soon as possible, sub‑fertile ejaculates from use in artificial insemination programs.

One of the main limitations in biomarker compilation is the inherent bias arising from differences in experimental approaches, limited sample sizes, breed and age effects, and the use of diverse bioinformatic algorithms for –omics analyses, among other factors. Therefore, it is essential to exercise caution when proposing a single, universal sperm quality or fertility biomarker. Instead, a balanced integration of conventional parameters (sperm motility and kinematics, morphology, membrane integrity), advanced –omics data, GWAS findings, and multiparametric bioinformatic evaluation is required.

From a physiological perspective, the classical DNA-RNA-PROTEIN smooth pathway is largely carried out nowadays by extremely small yet well-proven signalling molecules, the so-called EVs. In our opinion, these EVs are the future (and already the present (Martín-San Juan et al., 2026)) of sperm quality/fertility discovery. With that in mind, our research perspective should focus not only on advancing biological knowledge but also on providing stakeholders with valuable fertility predictors.

## Data Availability

Research data is only available upon request.
